# Genetic Analysis of a Novel Tubulin Mutation That Redirects Synaptic Vesicle Targeting and Causes Neurite Degeneration in *C. elegans*

**DOI:** 10.1371/journal.pgen.1004715

**Published:** 2014-11-13

**Authors:** Jiun-Min Hsu, Chun-Hao Chen, Yen-Chih Chen, Kent L. McDonald, Mark Gurling, Albert Lee, Gian Garriga, Chun-Liang Pan

**Affiliations:** 1 Institute of Molecular Medicine, College of Medicine, National Taiwan University, Taipei, Taiwan; 2 Electron Microscope Laboratory, University of California, Berkeley, Berkeley, California, United States of America; 3 Department of Molecular and Cell Biology and Helen Wills Neuroscience Institute, University of California, Berkeley, Berkeley, California, United States of America; 4 Department of Chemistry, National Taiwan University, Taipei, Taiwan; University of California, San Diego, United States of America

## Abstract

Neuronal cargos are differentially targeted to either axons or dendrites, and this polarized cargo targeting critically depends on the interaction between microtubules and molecular motors. From a forward mutagenesis screen, we identified a gain-of-function mutation in the *C. elegans* α-tubulin gene *mec-12* that triggered synaptic vesicle mistargeting, neurite swelling and neurodegeneration in the touch receptor neurons. This missense mutation replaced an absolutely conserved glycine in the H12 helix with glutamic acid, resulting in increased negative charges at the C-terminus of α-tubulin. Synaptic vesicle mistargeting in the mutant neurons was suppressed by reducing dynein function, suggesting that aberrantly high dynein activity mistargeted synaptic vesicles. We demonstrated that dynein showed preference towards binding mutant microtubules over wild-type in microtubule sedimentation assay. By contrast, neurite swelling and neurodegeneration were independent of dynein and could be ameliorated by genetic paralysis of the animal. This suggests that mutant microtubules render the neurons susceptible to recurrent mechanical stress induced by muscle activity, which is consistent with the observation that microtubule network was disorganized under electron microscopy. Our work provides insights into how microtubule-dynein interaction instructs synaptic vesicle targeting and the importance of microtubule in the maintenance of neuronal structures against constant mechanical stress.

## Author Summary

Axons and dendrites are two classes of neuronal process that differ in their functions and molecular compositions. Proteins important for synaptic functions are mostly synthesized in the cell body and sorted differentially into the axon or dendrites. Microtubules in the axon and dendrite maintain their structural integrity and regulate polarized protein transport into these compartments. We identified a novel α-tubulin mutation in *C. elegans* that caused mistargeting of synaptic vesicles and induced progressive neurite swelling, which resulted in late-onset neurodegeneration. We showed that this tubulin mutation weakened microtubule network and abnormally increased microtubule affinity for dynein, a motor protein responsible for cargo sorting to the dendrite. This enhanced microtubule-dynein affinity is due to augmented negative charge at the carboxyl terminus of α-tubulin. Neurite swelling and neurodegeneration could be ameliorated by reduced physical activity, suggesting that recurrent mechanical strain from muscle contraction jeopardized neurite integrity in the long run. Mutations in α- and β-tubulins are found in human neurological diseases; our findings therefore contribute to understanding the pathogenic mechanism of human neurological diseases associated with tubulin mutations.

## Introduction

Microtubule and molecular motors mediate polarized transport of neuronal proteins to either axons or dendrites [1]. Microtubules are oriented uniformly with their plus ends towards the distal end of the axon, which facilitates kinesin-dependent targeting of presynaptic proteins [2]. By contrast, targeting of postsynaptic molecules to the dendrite, such as glutamate receptors, requires the minus end-oriented dynein motors [3], consistent with the fact that many microtubules in the dendrites orient the minus end distally [4]. Mislocalization of presynaptic proteins to the dendrite occurs when this polarized pattern of axon-dendritic microtubule arrays is disrupted [5], [6], when kinesin function is compromised [7], [8], or when dynein activity is inadvertently increased [3], [7], [8]. Synaptic vesicle (SV) precursors are generated in the neuronal cell body and transported to the synapses by the unidirectional motor Kinesin 3/KIF1A [1]. On the other hand, the dynein motor complex mediates retrograde SV transport in the axon [1], [9]. Since SVs are cargos for both KIF1A and dynein, it is intriguing that they are exclusively targeted to the axon and prevented from entering the dendrites.

Previous biochemical and structural studies suggest that kinesin and dynein share an overlapping binding region at the C-terminus of α-tubulin [10]. The N-terminus of the H12 helix of the α-tubulin contains a stretch of absolutely conserved acidic residues (414EEGE, equivalent to 415EEGE in the yeast α-tubulin) and interacts with ATP-bound KIF1A [11]. A recent study on dynein structures also implicates this region in the interaction between microtubule and the microtubule-binding domain (MTBD) of dynein [12], although validation of this model in the context of in vivo, eukaryotic system is still lacking. Mutations of any of the three glutamic acids in the yeast α-tubulin to alanine dramatically reduced the frequency of kinesin binding to the microtubules [13]. Mutations of several conserved, acidic residues in the H12 helix of β-tubulin (E410, E412, D417) to alanine similarly reduced microtubule affinity for kinesins. Interestingly, E410K, D417H and D417N in the human β-tubulin *TUBB3*, among other point mutations, had been found in patients with congenital neurological syndrome with ophthalmoparesis and peripheral neuropathy [14]. In particular, TUBB3(E410K) and TUBB3(D417H), but not other disease-related TUBB3 mutations, were shown to impair the bindings of Kinesin 1/KIF5, Kinesin 3/KIF1A and KIF21 when expressed in cultured mammalian neurons [15]. The interaction between dynein and microtubule was not affected by these TUBB3 mutants [15]. These studies established the critical importance of negative charge in the H12 helix of the α- and β-tubulins in mediating microtubule-kinesin interaction, but the molecular mechanisms governing microtubule-dynein interaction and its physiological significance remain unexplored.

Here we describe a novel mutation of G416 in the α-tubulin MEC-12 of *C. elegans* to glutamic acid (G416E). Homologous mutations at this site of α-tubulin had not been reported in human diseases or tested in genetic model organisms. In *C. elegans*, *mec-12* is highly expressed in the six touch receptor neurons that detect gentle mechanical stimulation on the worm cuticle [16], [17]. MEC-12 and the touch neuron-specific β-tubulin MEC-7 are required to form the unusual, 15-protofilament giant microtubules in these neurons [16]. These giant microtubules had been implicated in the transduction of mechanosensation, although the mechanisms remain enigmatic [18]. We show that this gain-of-function G416E mutation redirects SVs to non-axon compartment in the *C. elegans* mechanosensory neuron PLM, and it does so by increasing microtubule affinity for dynein.

## Results

### The *gm379* mutation disrupted synaptic vesicle transport and caused synaptic vesicle mistargeting

**Figure 1:**
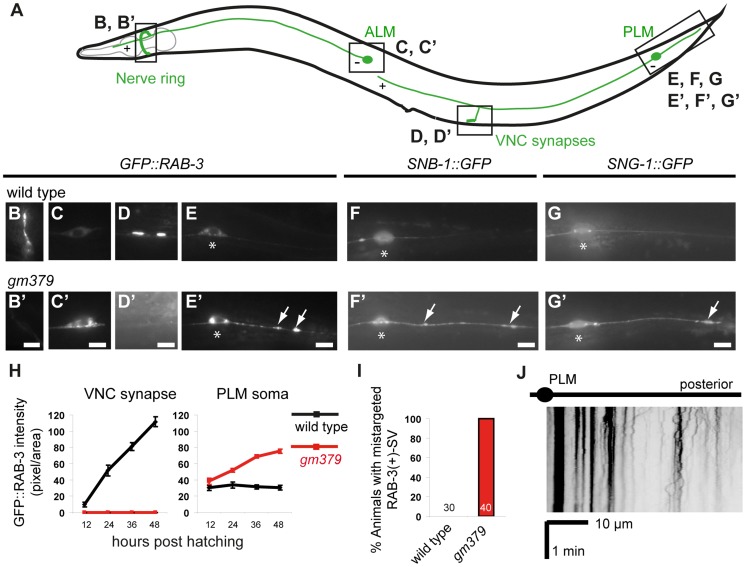
Synaptic vesicle mistargeting and transport defects in the *gm379* mutant. (A) A schematic diagram of the *C. elegans* ALM and PLM neurons and their synapses. The “+” and “−“ signs indicate the dominant microtubule orientation in the anterior ALM and PLM processes. (B-G, B′-G′) Synaptic vesicles in live animals were visualized and quantified with *jsIs821(Pmec-7::GFP::RAB-3)* (B-E, B′-E′) from the nerve ring synapses (B, B′), ALM soma (C, C′), PLM synapses in the ventral nerve cord (D, D′), PLM soma (E, E′, asterisks) and the PLM posterior process (E, E′). SV mistargeting was independently verified with *jsIs37(Pmec-7::SNB-1::GFP)* (F, F′) and *jsIs219(Psng-1::SNG-1::GFP)* (G, G′). Arrows, mistargeted SVs. Scale bar  = 5 µm. (H) SV abundance measured by GFP::RAB-3 quantification (mean ± S.E.M.) in respective locations of the touch neuron circuit. N = 10 animals for each genotype at each time point. (I) Percentage of animals with SV mistargeting. (J) Kymograph of SV in the PLM posterior process of the mutant.

In wild-type *C. elegans*, the bilaterally symmetric touch receptor neurons ALM and PLM develop a single anterior process that forms synapses in the nerve ring and in the ventral nerve cord, respectively (Figure 1A). Touch neuron synapses are enriched in RAB-3-(+) synaptic vesicles (SVs), the active zone protein SYD-2/Liprin-α, and mitochondria (Figure 1B, 1D, and S1) [19], [20]. The PLM neurons also have a short posterior process that does not form synapses. In an EMS mutagenesis screen (see Materials and Methods), we identified *gm379*, a mutant with prominent SV phenotypes in the touch neurons (Figure 1B′-1G′). *gm379* animals lacked RAB-3-(+) SVs at the touch neuron synapses, and instead SVs accumulated in the neuronal soma (Figure 1B′-1E′, 1H). We refer to these as SV transport defects for the rest of the paper. Surprisingly, SVs were also redirected to the PLM posterior process, a phenotype that we call SV mistargeting (Figure 1E′, 1I). These results were confirmed using two other SV reporters, *jsIs37(Pmec-7::SNB-1::GFP)* that marked the SV membrane protein synaptobrevin/SNB-1, and *jsIs219(Psng-1::SNG-1::GFP)* labeling another SV protein synaptogyrin/SNG-1, with GFP (Figure 1F-1G′). These ectopic SVs showed very limited motility, and many were stationary (Figure 1J). We followed *gm379* mutants through development, and confirmed that SV transport defects and mistargeting were present at early larval stages and progressively worsened (Figure 1H). The transport and targeting of synaptic active zone protein SYD-2 was affected to a much milder degree, and surprisingly, SYD-2 failed to mistarget to the PLM posterior process (Figure S1). The dissociation in the mutant phenotypes of SV and active zone proteins indicates that the *gm379* mutation caused relatively specific defects in SV targeting rather than generally impaired axon transport or induced ectopic synapse formation.

### The *gm379* mutant developed microtubule disorganization, neurite swelling and degeneration of the touch neurons

**Figure 2:**
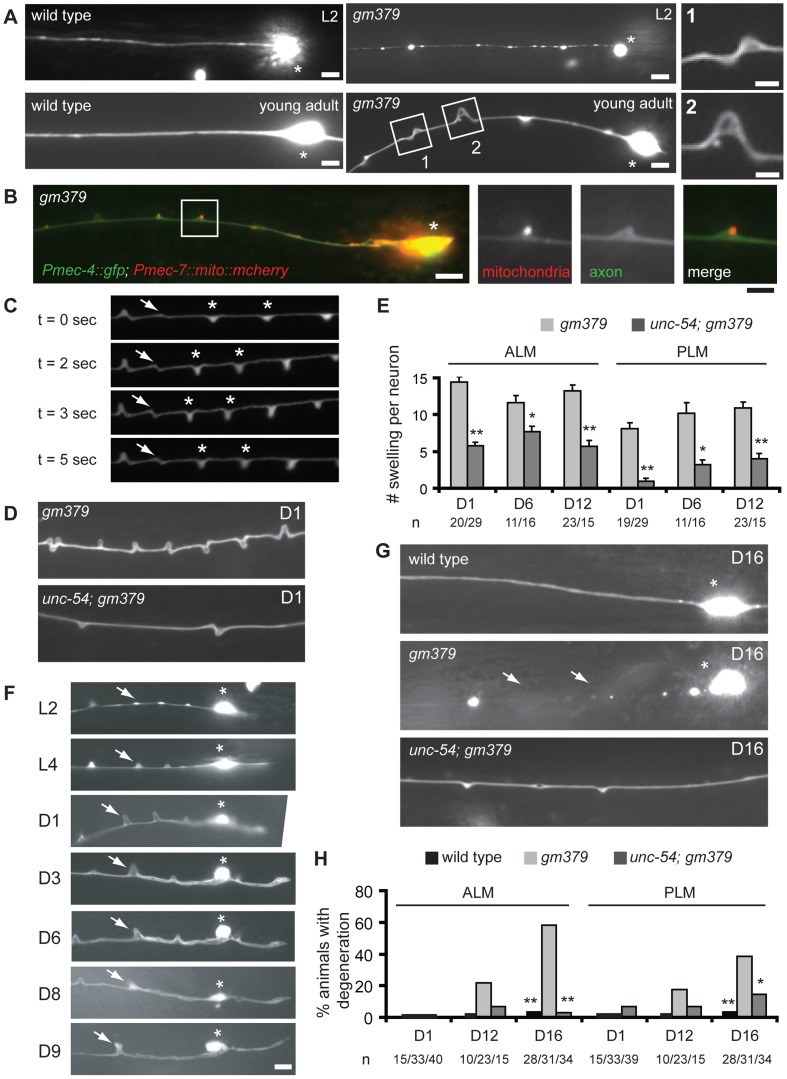
Axon pathology of the touch neurons in the *gm379* mutant. (A-B) Touch neurons in live animals were visualized with *zdIs5(Pmec-4::GFP)*. Asterisks, ALM soma. Scale bar  = 5 µm or 2 µm (insets). (A) ALM processes in the mutant appeared beaded at L2 and developed triangular-shaped swelling at L4 to adult. Anterior is to the left. (B) Mitochondria, labeled with *Pmec-7::mito::mCherry*, often accumulated at axon swellings. (C) Snapshots of PLM neurite during muscle contraction from a *gm379* mutant partially immobilized with microbeads. Arrows and asterisks are buckling and neurite swellings, respectively, that changed their morphology during muscle contraction. Muscles contracted between t  = 2 second and t  = 5 second. (D) ALM neurites of the *gm379* or the *unc-54; gm379* mutants. (E) Quantification (mean ± S.E.M.) of neurite swellings for single ALM or PLM neurons. Age of the animals was indicated as days in adulthood. *, *p*≤0.01, **, *p*<0.0001, Mann-Whitney U test. (F) Longitudinal images of a single ALM neuron from L2 to middle adulthood. Arrows marked the same site over time. The animal died at day 10. Scale bar  = 5 µm. Asterisks, ALM soma. (G) ALM neurites of the wild type, *gm379* and the *unc-54; gm379* mutants at day 16. Asterisks are ALM soma. Arrows mark neurite breaks and degeneration. (H) Quantification (mean ± S.E.M.) of neurodegeneration. *, *p*<0.05, **, *p*<0.0001, Fisher's exact test.

In addition to SV transport defects, the *gm379* mutant touch neurons had progressive neurite swelling and misshapen soma (Figure 2A and S2A). Neurite defects evolved from small beadings at early larval stages into triangular-shaped swellings in L4 and adult animals, and mitochondria were frequently found to be present at the swellings (Figure 2A, 2B and S2A). These swellings were dynamic in morphology, as movements often induced reversible buckling of the neurite and changed the width and height of the swellings (Figure 2C and Video S1), which was similar to what had been described earlier for the tubulin acetyltransferase mutant *mec-17* [21]. Neurite buckling or swelling were never seen in the wild type even under maximal muscle contraction induced by levamisole. This observation suggests that the *gm379* mutation rendered the touch neurite susceptible to deformation under mechanical strain, a phenotype that was also seen when the membrane skeleton protein UNC-70/β-spectrin was lost [22]. We therefore test whether genetic paralysis of the animals suppresses neurite defects of the *gm379* mutant. Mutation in the muscle myosin gene *unc-54* almost completely paralyzed the animals, and it significantly reduced the number of neurite swellings in the *gm379* mutant touch neurons (Figure 2D and 2E). This result implies that the *gm379* mutation compromises the ability of the touch neurites to cope with mechanical stress.

We were curious whether massive neurite swellings in the *gm379* mutant predispose touch neurons to degeneration. We first performed longitudinal imaging of individual ALM and PLM neurons through adulthood, and found that touch neurites in the *gm379* mutant underwent progressive disorganization (Figure 2F). Touch neuron degeneration, characterized by swelling of neuronal soma, neurite interruption and extensive beadings, began to emerge in the *gm379* mutant at D9 and progressively increased (Figure 2G and 2H). Touch neuron degeneration was extremely rare in the wild type at comparable age (Figure 2G and 2H) [23]. Interestingly, touch neuron degeneration of the *gm379* mutant was suppressed by the *unc-54* mutation (Figure 2G and 2H). These results indicate that recurrent mechanical strain imposed on the touch neurons during locomotion is an important precipitating factor for late-onset neurodegeneration in the *gm379* mutant.

**Figure 3:**
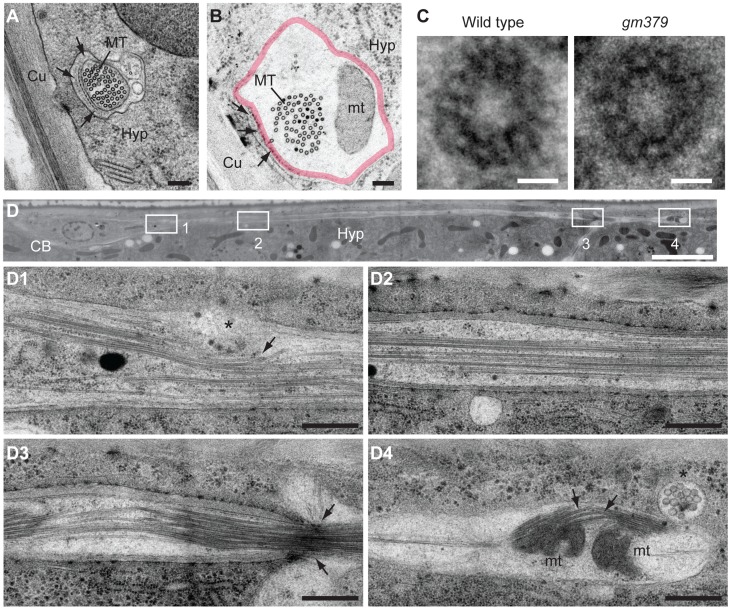
Ultrastructural characterization of neuronal defects of the *gm379* mutant. (A-C) Transverse EM sections showing the ultrastructure of touch neurons in the wild type (A and C, left panel) and in the mutant (B and C, right panel). (A, B) Microtubule numbers within the touch neuron process were preserved in the mutant. However, a mitochondrion (mt) was located eccentrically, resulting in a marked increase in focal axon diameter (marked by the thick red line). The extracellular matrix (arrows) that normally surrounds the touch neuron process seemed to break at axon swelling sites. Scale bar  = 150 nm. (C) Individual microtubule polymers from the wild type (left) and the mutant (right) both contained 15 protofilaments. The intraluminal electron-dense materials in the microtubule were also preserved in the mutant. Scale bar  = 10 nm. (D) Longitudinal EM section of an ALM neuron in the adult mutant, with anterior to the right. Scale bar  = 5 µm. (D1) Microtubule bundles were compressed (arrow) by some organelles of uncertain identity (asterisk) at the proximal axon segment. (D2) Microtubule polymers assumed straight, more wild-type appearance at certain regions of the axon. (D3) Microtubules were compressed and strangled at focal axon constriction (arrow), which profoundly distorted the axonal cytoskeleton. (D4) Focal mitochondrial accumulation (mt) distorted microtubule bundles (arrows), the latter assuming a curved configuration that was never seen in the wild-type axon. A small protrusion (asterisk) from the axonal membrane contained small membrane-bound profiles of unknown identity. Scale bars  = 0.5 µm. CB, cell body of ALM; Cu, cuticle; Hyp, hypodermal cell; MT, microtubules.

Under serial thin-section electron microscopy, we found that the characteristic 15-protofilament microtubules of *C. elegans* touch neurons were preserved in the *gm379* mutant, including those in the PLM posterior process (Figure 3A-C, S2B-D). Mitochondria could be found where touch neuron processes swelled abnormally (Figure 3B), consistent with our light microscopic observation (Figure 2B). In longitudinal sections, in contrast to the wild type, where neuronal microtubules formed long straight bundles, touch neuron microtubules in the *gm379* animals curved focally at sites of organelle accumulation (Figure 3D). We observed bending and splitting of neuronal microtubule bundles at sites of mitochondria accumulation in focal axonal swellings (Figure 3D4). These ultrastructural studies suggest that the microtubule network of the touch neurons is abnormal in the *gm379* mutant.

### *gm379* is a neomorphic allele of the α-Tubulin *mec-12*

**Figure 4:**
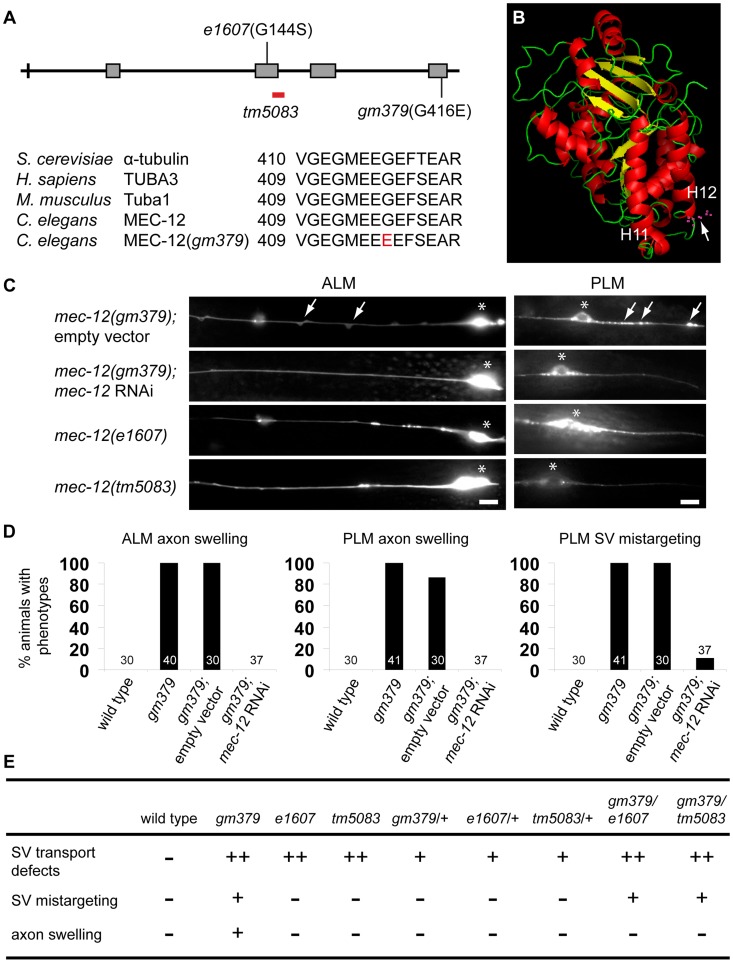
Genetic analysis of *gm379* and other *mec-12* alleles. (A) The gene structure and mutant alleles of *mec-12*, and comparison of α-tubulins in yeast, *C. elegans*, mice and human. Characters and numbers are nucleotides and their cDNA positions (upper panel) or amino acids (lower panel). (B) Predicted protein structure of MEC-12 generated by Polyview software. The G416E mutation in *gm379* is located in the H12 helix. (C, D) RNAi against *mec-12* completely suppressed axon defects and largely abolished SV mistargeting in the *gm379* mutant, phenocopying the *mec-12(e1607)* and *mec-12(tm5083)* null mutants. Neurons and SVs were labeled by *jsIs973* and *jsIs821*, respectively. Arrows indicate axon swellings in ALM and mistargeted SVs in PLM. Asterisks indicate neuronal soma. Anterior is to the left. Scale bars  = 5 µm. (E) Genetic analysis of various *mec-12* alleles. -, no phenotypes; +, moderate defects; ++, prominent defects.

We cloned *gm379* by single nucleotide polymorphism (SNP) mapping, complementation test and DNA sequencing, and found that it contained a missense mutation of the touch neuron-specific α-tubulin *mec-12* that alters an absolutely conserved C-terminal glycine residue to glutamate (G416E) [19], [17] (Figure 4A and 4B). Interestingly, *gm379* animals were touch-insensitive (30% touch-sensitive, compared to wild type, 89%; and *mec-12(e1607)* null, 12%, n>35). We found another intron mutation in *gm379* that was distant to exon-intron junctions (nucleotide 828 of unspliced transcript, G to A mutation). Two null mutants of *mec-12*, *e1607* and *tm5083*, also had SV transport defects, but not SV mistargeting in the PLM or axon swelling in the touch neurons (Figure 4C, S3). RNAi against *mec-12* in the *gm379* mutant almost completely abolished axon swelling or SV mistargeting, indicating that these two phenotypes were neomorphic (Figure 4C, 4D). Expression of the MEC-12(G416E) mutant tubulin in the *mec-12* null mutants recapitulated the SV mistargeting phenotypes of the *mec-12(gm379)* mutant (67% of transgenic animals showed SV mistargeting, n = 21 v.s. 6.5% of array-loss siblings showing SV mistargeting, n = 77), confirming that SV mistargeting and neurite swelling were indeed caused by the *mec-12(gm379)* rather than other unidentified mutations in the background.

To gain further insights into the genetic nature of the *mec-12* phenotypes, we analyzed various heterozygous *mec-12* mutants as well as trans-heterozygotes between these alleles (Figure 4E). Heterozygous animals containing the *e1607*, *tm5083* or *gm379* mutations all showed moderate SV transport defects that were less severe than the homozygous animals. These observations suggest that loss of *mec-12* functions causes semi-dominant SV transport defects due to haploinsufficiency. Heterozygous *gm379* mutants did not display neurite swelling or SV mistargeting. By contrast, *mec-12(gm379)/mec-12(e1607)* and *mec-12(gm379)/mec-12(tm5083)* trans-heterozygous mutants had SV mistargeting without neurite swelling, implying that the presence of wild-type MEC-12 somehow prevents MEC-12(G416E) from mistargeting SVs or disrupting microtubule organization. In conclusion, our genetic analysis indicates that *gm379* causes both neomorphic (neurite swelling and SV mistargeting) and semi-dominant loss-of-function (SV transport defects) phenotypes of *mec-12*.

### SV mistargeting in *gm379* mutant was independent of microtubule polarity, tubulin posttranslational modifications or UNC-104/KIF1A

Stable microtubules formed in the *gm379* touch neurons, based on the EM data and the preserved lysine 40 (K40) acetylation of α-tubulin [17], [24], [25] (Figure S4A). Moreover, a *mec-7*/β-tubulin null mutation completely suppressed neurite swelling and dramatically reduced SV mistargeting of *mec-12(gm379)* (Figure S5), suggesting that SV mistargeting and neurite swelling phenotypes require intact microtubules. Labeling touch neuron microtubules with the plus end-binding protein EBP-2::GFP showed that in the wild type, microtubules oriented plus-end distally in the anterior PLM process (Figure S6) [26]. In the PLM posterior process, microtubules showed mixed polarity (Figure S6). These patterns of microtubule polarity were preserved in the *mec-12(gm379)* mutant (Figure S6).

A recent study reported that mutations in the tubulin acetyltransferase *mec-17* caused neurite degeneration with SV mislocalization in the touch neurons [27]. To test whether altered microtubule posttranslational modifications are responsible for SV mistargeting in the *mec-12(gm379)* mutant, we performed immunostaining experiments, but did not observe gross difference in microtubule acetylation or tyrosination in the touch neurons between the wild type and the *mec-12(gm379)* mutant (Figure S4A). Tubulin polyglutamylation signal was restricted to the amphid and phasmid sensory cilia as in the wild type, and was not ectopically expressed in the mutant touch neurons (Figure S4B). Moreover, we performed feeding RNAi to knock down the tubulin polyglutamylase *ttll-4*, the tubulin deglutamylase *ccpp-6*, and a few genes (*ttll-5*, *ttll-12*, *ttll-15* and *ccpp-1*) that bear sequence homology to human tubulin amino acid ligases (Wormbase at http://www.wormbase.org) [28], [29], in both wild type and the *mec-12(gm379)* mutant. None of these RNAi resulted in SV mistargeting in the wild type or suppressed SV mistargeting in the *mec-12(gm379)* animals. Based on these results, we conclude that SV mistargeting in the *mec-12(gm379)* is not a consequence of altered microtubule posttranslational modifications.

**Figure 5:**
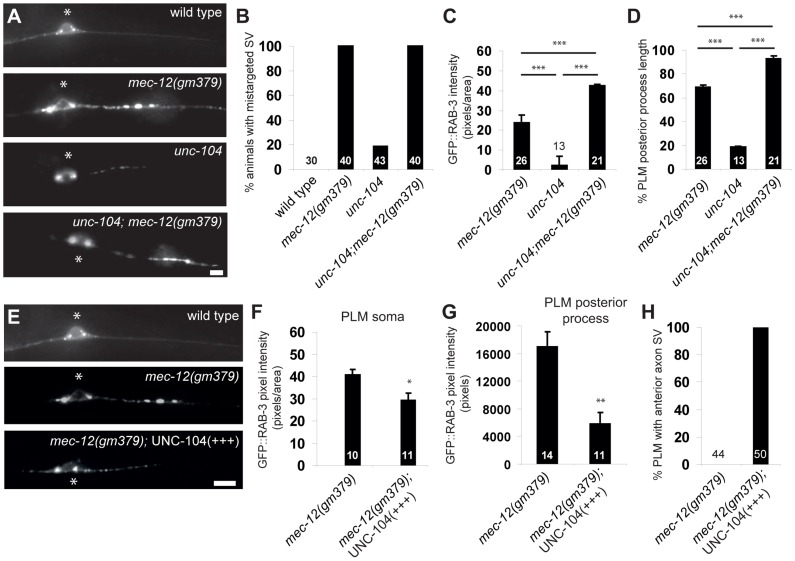
Genetic interaction between *mec-12(gm379)* and *unc-104*. (A-D) *unc-104* mutations aggravated SV mistargeting in the *mec-12(gm379)* mutant. GFP is *jsIs821(Pmec-7::GFP::RAB-3)*. (A) Epifluorescence images of the PLM cell body and the posterior process. Anterior is to the left. Asterisks, PLM cell body. Percentage of animals with SV mistargeting (B), the abundance of mistargeted SV (C) and the distribution of mistargeted SV (D), which was the most distal GFP::RAB-3 distribution as a percentage of the total length of the PLM posterior process. Quantifications are mean ± S.E.M. (E-H) UNC-104 overexpression rescued SV mistargeting in the *mec-12(gm379)* mutant. Epifluorescence images of the PLM with the same parameters as those in (A). With excess UNC-104, SVs accumulated in the PLM soma (F) or mistargeted to the PLM posterior process (G) were decreased, and they were able to enter the anterior PLM process again (H). Quantifications are mean ± S.E.M. Scale bars  = 5 µm. *, *p*<0.05; **, *p*<0.001; ***, *p*<0.0001, Mann-Whitney U test.

It is possible that the interaction between KIF1A and microtubule was altered by the G416E mutation. We found that the strong loss-of-function *unc-104(rh43)*/KIF1A mutation caused completely penetrant SV transport defects and, surprisingly, low percentage of SV mistargeting in the PLM (Figure 5A and 5B). Moreover, this *unc-104* mutation enhanced SV mistargeting of *mec-12(gm379)* rather than suppressing the phenotype, with more SVs mistargeted to the PLM posterior process and distributed more distally (Figure 5C, 5D). This result suggests that SV mistargeting in the *mec-12(gm379)* mutant is not caused by aberrant UNC-104 activity. Furthermore, overexpression of UNC-104 significantly rescued SV transport defects and mistargeting in the *mec-12(gm379)* mutant, with more SVs reaching synapses in the nerve ring or entering the anterior ALM and PLM processes (Figure 5E-5H, Figure S7A, S7B). While these data are consistent with the interpretation that UNC-104 activity was reduced in the *mec-12(gm379)* mutant, resulting in severe SV transport defects, they also indicate that SV mistargeting in the mutant requires the activity of an unknown molecule.

### SV mistargeting in the *gm379* mutant requires DHC-1/Dynein

**Figure 6:**
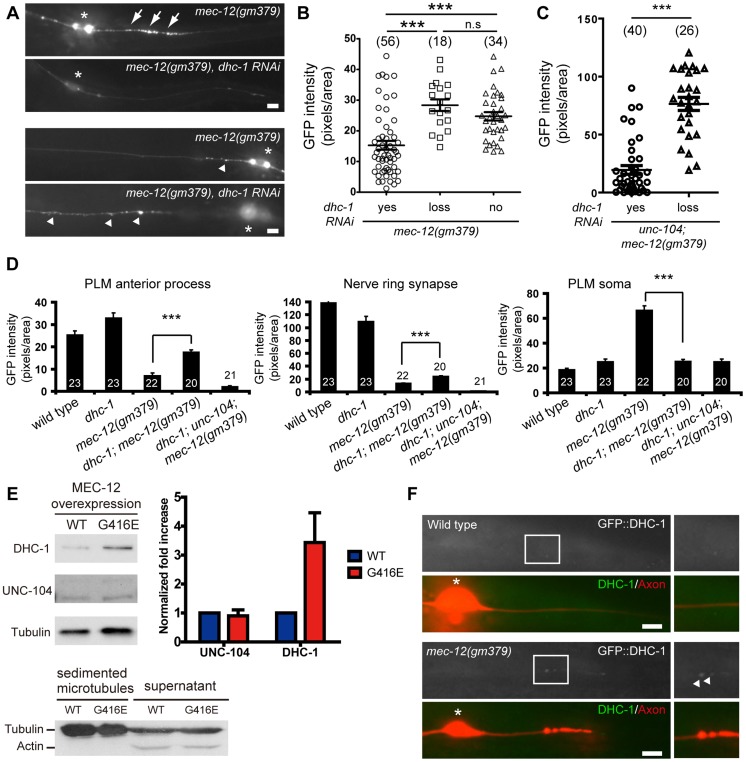
SV mistargeting in the *mec-12(gm379)* mutant depends on dynein activity. (A) SV distribution in the PLM posterior process (upper panels) and the anterior PLM process (lower panels) visualized by *jsIs821(Pmec-7::GFP::RAB-3)*. Anterior is to the left. Normal and mistargeted SVs were labeled by arrowheads and arrows, respectively. Asterisks, PLM soma. (B-C) Quantification of SV mistargeting in the *mec-12(gm379); twnEx42[Pmec-7::dhc-1(sense/antisense)]* (B) or in the *unc-104; mec-12(gm379); twnEx42* (C) animals. *yes*, animals with the array expressed in the PLM; *loss*, transgenic animals with the array lost from the PLM; *no*, non-transgenic animals. (D) Quantification (mean ± S.E.M.) of SV density upon loss of DHC-1 functions in the *mec-12(gm379)* mutant. (E) Microtubule sedimentation assay. Microtubules were sedimented from animals overexpressing wild-type MEC-12 or MEC-12(G416E), and probed with antibodies against UNC-104 or DHC-1. Immunoreactivity for 6-11B-1 confirmed the presence of MEC-12 in the samples. Actin was present in the supernatant but not in the sediment, suggesting that the sedimentation procedure was highly successful. n = 3 for each experiment. Quantification (mean ± S.E.M.) and normalization of band intensity were described in Materials and Methods. (F) Epifluorescence images showing the recruitment of GFP::DHC-1 (arrowheads) to the PLM posterior process of the *mec-12(gm379)* but not the wild-type animals. Boxed regions were highlighted on the right. Axons were marked by *jsIs973(Pmec-7::mRFP)*. Asterisks, PLM soma. Scale bars  = 5 µm. ***, *p*<0.0001, Mann-Whitney U test.

We wondered whether increased activity of the minus end motor dynein is responsible for SV targeting to the PLM posterior process in the mutant, based on the presence of minus end-out microtubules in the PLM posterior process and the *unc-104* effects. *dhc-1* encodes the heavy chain for cytoplasmic dynein in *C. elegans* [30]. If enhanced dynein activity is responsible for SV mistargeting in the mutant, elimination of dynein function should suppress it. We could observe SV mistargeted to the PLM posterior process as early as 2-3 fold embryos, before the animal hatched. With the available *dhc-1* mutant alleles, it was not possible to lose DHC-1 functions at such early stages without compromising animals' viability. To eliminate DHC-1 functions as early as possible, and to circumvent lethality due to widespread DHC-1 loss, we specifically knocked down *dhc-1* in the touch neurons, but not in other somatic tissues, by simultaneously expressing sense and antisense *dhc-1* from the *mec-7* promoter, which we named transgenic *dhc-1* RNAi. Strikingly, transgenic *dhc-1* RNAi significantly suppressed SV mistargeting of the *mec-12(gm379)* mutant, with about one third of the transgenic animals completely devoid of mistargeted SVs (Figure 6A, 6B). This result was confirmed by another independently generated *dhc-1* RNAi array (Figure S8A). Transgenic *dhc-1* RNAi also significantly reduced SV mistargeting in the *unc-104; mec-12(gm379)* mutant (Figure 6C). In the wild type, transgenic *dhc-1* RNAi had little effects on the intensity of GFP::RAB-3 or SNB-1::GFP in the PLM soma or synapses (Figure S8B). These data indicate that SV mistargeting in the *mec-12(gm379)* mutant is mediated by the dynein motor. The neurite swelling phenotypes of the mutant, by contrast, were not changed by *dhc-1* RNAi, suggesting that SV mistargeting and neurite defects are mechanistically distinct.

We noted that eliminating DHC-1 functions in the *mec-12(gm379)* mutant significantly restored synaptic targeting and anterograde transport of SVs, with concomitant reduction of SV accumulation in the touch neuron soma (Figure 6A and 6D). These effects were similar to those caused by excess UNC-104 (Figure 5E-H, S7A, S7B). Indeed, synaptic targeting and anterograde transport of SVs were completely abolished in the *dhc-1; unc-104; mec-12(gm379)* triple mutant, suggesting that the phenotypic rescue caused by the *dhc-1* mutation requires UNC-104 (Figure 6D). Although significantly increased compared to those in the *mec-12(gm379)* mutant, SV signals in the nerve ring synapses of the *dhc-1; mec-12(gm379)* were still much weaker than the wild type. Together with the severe reduction of presynaptic SVs in the *mec-12(gm379)* mutant, these observations suggest that UNC-104 was not able to display a full range of activity on the mutant microtubule scaffolds.

### MEC-12(G416E) microtubules had increased affinity for dynein

To test whether the MEC-12(G416E) mutant microtubules have increased affinity for dynein, we performed microtubule sedimentation in transgenic strains expressing MEC-12 and MEC-7 pan-neuronally, and assayed for the amount of DHC-1 or UNC-104 associated with microtubules by blotting with anti-DHC-1 and anti-UNC-104 antibodies, respectively. The presence of MEC-12-containing, stable microtubules was verified by detecting 6-11B-1 antibody immunoreactivity in neurons other than the touch receptors (). After normalization to tubulin, the amount of DHC-1 co-sedimented with MEC-12(G416E)-containing microtubules dramatically increased, compared to that co-sedimented with wild-type microtubules (Figure 6E). In support of this conclusion, we found that GFP::DHC-1, expressed from the low-copy integrated transgene *orIs17(Pdhc-1::gfp::dhc-1)*, which drives DHC-1 expression from the endogenous promoter, was accumulated in the PLM posterior process in the *mec-12(gm379)*, but not in the wild type (Figure 6F). Together these results indicate that mutant microtubules have increased affinity for DHC-1. To our surprise, we did not detect a change in the amount of UNC-104 co-sedimented with mutant microtubules (Figure 6E) or a change of UNC-104 protein localization in the PLM neuron. This implies that UNC-104 can still associate with the mutant microtubules, although its function is somehow disrupted.

### Negative charges at the H12 helix of MEC-12 instruct SV targeting

**Figure 7:**
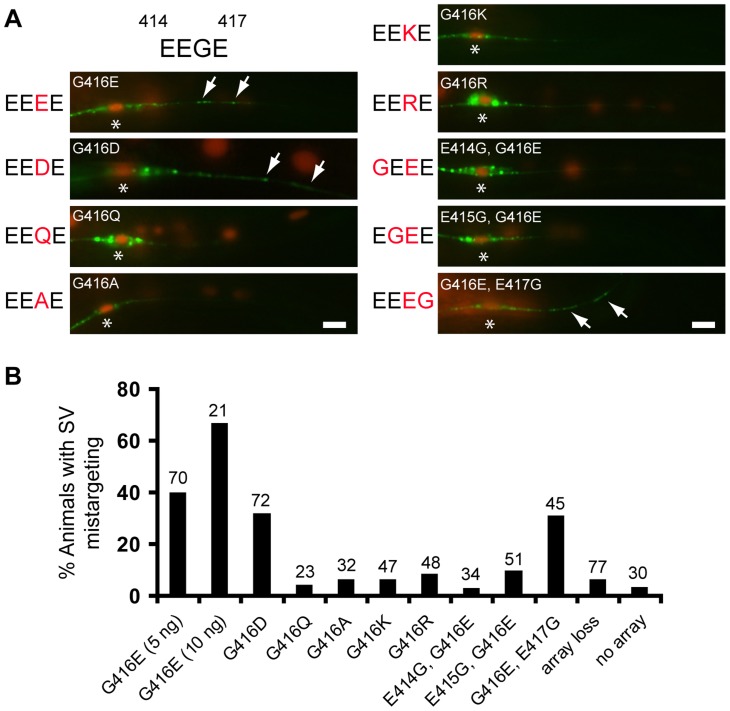
Negative charges of the EEGE cluster in the MEC-12 H12 helix instruct synaptic vesicle targeting. (A) Effects of G416 substitution on SV targeting in the PLM neuron. Expression of respective MEC-12 variants in the *mec-12(e1607)* was indicated by the cell-autonomous marker *Pdpy-30::NLS::dsRed*. SVs were labeled by *jsIs821(Pmec-7::GFP::RAB-3).* Arrows, mistargeted SVs. Asterisks, PLM soma. Scale bar  = 5 µm. (B) Quantification of SV mistargeting in animals expressing MEC-12 variants. Array loss, transgenic animals that lost the MEC-12-expressing array from the PLM. No array, non-transgenic animals. Scale bars  = 5 µm.

The aforementioned data indicate that G416 of MEC-12 plays a critical role in determining the relative affinity of microtubules for dynein. To further decipher the mechanisms that instruct microtubule-dynein affinity, we systemically replaced G416 with acidic (aspartic acid/D) or basic (lysine/K, arginine/R) residues, as well as alanine (A) and glutamine (Q), the latter being similar to glutamic acid in side chain length but did not carry charges (Figure 7A, 7B). These MEC-12 species were expressed in the touch neurons of the *mec-12(e1607)* null mutant. SV mistargeting was seen only with the expression MEC-12(G416D), but not other G416 substitutions (Figure 7A, 7B). These results suggest that SV mistargeting in the *gm379* mutant was caused by the increased negative charges at the EEGE cluster of MEC-12.

Is the arrangement of the acidic residues important for dynein affinity of microtubules? To answer this question, we moved the glycine to residue 414, 415 or 417, with reciprocal glutamic acid substitution at 416 (E414G/G416E, E415G/G416E, G416E/E417G, referred as GEEE, EGEE and EEEG, respectively; Figure 7A, 7B), so that the arrangement, but not the sum, of negative charges was altered, and asked whether this manipulation affects SV targeting. While expression of MEC-12(GEEE) or MEC-12(EGEE) did not result in significant SV mistargeting, expression of MEC-12(EEEG) triggered SV mistargeting in about 30% of the *mec-12(e1607)* animals (Figure 7A, 7B). These observations indicate that SV targeting critically depends on the magnitude and the spatial arrangement of negative charges in the EEGE cluster of the H12 helix.

### Mutation that disrupted intramolecular salt bridge in DHC-1 enhanced DHC-1 affinity with microtubules

**Figure 8:**
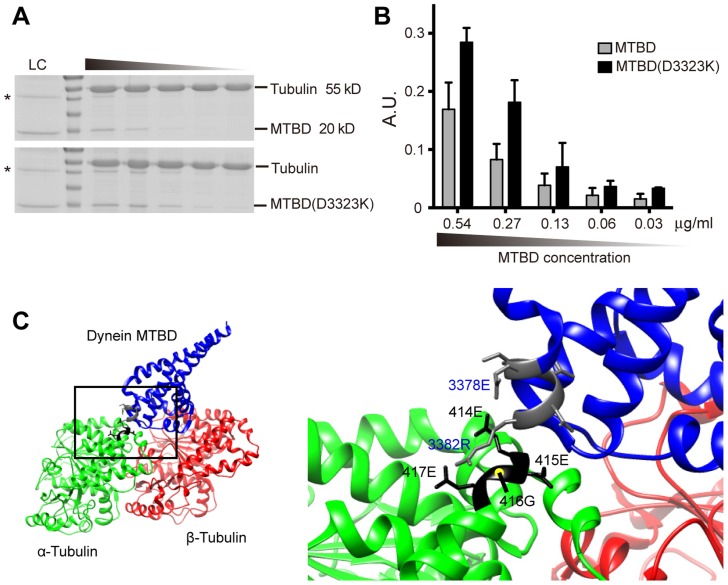
In vitro DHC-1 MTBD sedimentation assay and model of dynein MTBD-tubulin dimer interaction. (A) SDS-PAGE gel image stained with Coomassie blue for microtubule sedimentation using purified wild-type MTBD or MTBD(D3323K) of DHC-1. Lanes from left to right represent serial 2-fold dilution of MTBD loading, with concentrations indicated. LC, loading control. (B) Quantification (mean ± S.E.M.) of MTBD co-sedimented with microtubules. Data were averaged from three independent experiments. (C) The structure of the dynein microtubule-binding domain (MTBD) complexed with the tubulin dimer. Data from Protein Data Bank (PDB#3J1T) were processed by UCSF Chimera software. The interaction surface between the H12 of the α-tubulin and the dynein MTBD was highlighted in the bottom panel. Residues of the α-tubulin were labled in black, and those from the dynein MTBD were labeled in blue. An intramolecular salt bridge was suggested to form between E3378 and R3382 of the dynein MTBD when dynein is at some distance from the tubulin surface. Upon approaching the tubulin dimer, negative charges of the H12 of tubulin dimers were hypothesized to break the MTBD intramolecular salt bridge and foster the interaction between the MTBD and the tubulin dimer [12].

Previous structural studies suggest that dynein binds the H12 helix of α-tubulin [10], [12]. Redwine et al. proposed that E3378 and R3382 of the dynein microtubule-binding domain (MTBD) form intramolecular salt bridge, and upon approaching the tubulin dimer, negative charges of the α-tubulin H12 disrupt this salt bridge by attracting R3382, which carries positive charges (Figure 8C). An E3378K mutation of MTBD disrupted this intramolecular salt bridge and increased both the affinity and the run length of dynein on the microtubules [12]. We speculate that the E3378K mutation facilitates the electrostatic interaction between MTBD and the negative charges of the α-tubulin H12 domain. To test this, we expressed and purified a fragment of *C. elegans* DHC-1 MTBD, and showed that this MTBD precipitated with microtubules synthesized from purified bovine tubulin in the in vitro sedimentation experiment (Figure 8A and S9). Moreover, D3323K mutation, which is equivalent to E3378K mutation of the yeast dynein MTBD, enhanced MTBD-microtubule interaction across a range of tested concentrations (Figure 8A, 8B and S9). This result supports our hypothesis that exaggerated microtubule affinity for dynein critically depends on the electrostatic interaction between the EEGE-containing H12 helix of α-tubulin and the dynein MTBD (Figure 8C).

## Discussion

In the present study, we characterized a gain-of-function *mec-12*/tubulin mutant that displayed synaptic vesicle mistargeting and neurite swelling phenotypes, which were absent in the *mec-12* null mutant. In this mutant, single amino acid substitution augments microtubule-dynein interaction, thereby mistargeting synaptic vesicles to non-axon compartments. This observation extends previous structural studies on the charged cluster of the α-tubulin H12 helix and provides a biological context for such charge-based coupling between microtubule and dynein. The neurite swelling and degeneration phenotypes could bear important implications for human neurological diseases associated with missense tubulin mutations, as discussed below.

Previous structural studies suggest that dynein binds the H12 helix of α-tubulin, and the interaction between dynein and microtubule is not as strong as that between kinesins and microtubule [10], [12]. One advantage for this suboptimal dynein-microtubule affinity is flexibility and dynamic range for dynein processivity on the microtubule scaffolds. The hypothesis that dynein-microtubule interaction is not optimized also suggests that it is possible to further enhance dynein-microtubule affinity by mutating residues at this interface. Here, we show that this is indeed the case: by increasing negative charges of the α-tubulin H12 helix or augmenting positive charges of the dynein MTBD, microtubule-dynein interaction was strengthened. More importantly, we show that this aberrantly high affinity of microtubule for dynein redistributed synaptic vesicles to ectopic compartments in the neurons. We speculate that the suboptimal affinity of microtubule for dynein prevents initial SV entry into the dendrite, yet allows for retrograde SV transport within the axon. The G416E mutation of MEC-12 increased microtubule affinity for dynein probably at the expense of dynein processivity: we found that motility of the mistargeted SVs in the PLM posterior process was profoundly compromised (Figure 1J). Consistent with this view, Redwine et al. showed that E3378K mutation of MTBD caused about 40% reduction in the velocity of dynein movements on microtubules. By contrast, none of the G416 substitutions restored the UNC-104/KIF1A-dependent, anterograde SV transport in the *mec-12(e1607)* null mutant. This is also consistent with the observation by Redwine et al. that microtubule-kinesin interaction was molecularly optimized, therefore changes to the tubulin residues at the microtubule-kinesin interface will only compromise but not compensate for or even further improve this interaction.

Rather than merely serving as a track, mounting evidence indicates that microtubules play an active role in directing axon transport [2]. One such example is the various posttranslational modifications on microtubules, which regulate axon transport by restricting different kinesin motors to discrete subcellular compartments [31], [32]. The intrinsic organization of microtubule is another critical factor regulating polarized axon transport. UNC-33 and UNC-44, the *C. elegans* homologs for the Collapsin Response Mediator Protein 2 (CRMP2) and neuronal ankyrin, respectively [33], [34], regulate axon transport by maintaining uniform microtubule polarity in the axon and the dendrite [5]. In *C. elegans* head neurons, microtubules are uniformly oriented with their minus-ends towards the distal of the dendrite. In the *unc-33* and the *unc-44* mutants, dendritic microtubules showed mixed polarity, which resulted in aberrant sorting of axonal proteins into the dendrite [5]. In these mutants, the dendritic localization of SVs requires UNC-104. Of note, mistargeting of axonal proteins in the *unc-33* and *unc-44* mutants occurred to the SVs and multiple active zone components. By contrast, SYD-2 was not mistargeted in the *mec-12(gm379)* mutant, and we found that SV transport in the touch neurons was not affected in *unc-33* and *unc-44* mutants. These observations indicate that while perturbation to the gross architecture of microtubule causes extensive mistargeting of multiple axonal cargos, changes at a restricted, yet functionally critical site of microtubule could lead to targeting defects of specific axonal proteins.

Many tubulin mutations associated with human diseases are point mutations that alter protein function rather than eliminating protein products [35]–[37]. Mutant tubulins are still incorporated into microtubule polymers; phenotypes presumably arise from altered microtubule dynamics or disrupted interactions with molecular motors or microtubule-associated proteins [14], [15], [38]. The G416E mutation of MEC-12 caused extensive neurite swellings that eventually led to degeneration of the touch neurons. Ultrastructurally, there were unbundling of microtubules and accumulation of mitochondria at focal neurite swellings. Interestingly, these neurite defects were independent of dynein activity, but could be suppressed by genetic paralysis of locomotion. In light of two recent studies that showed touch neurite buckling or swelling when *unc-70*/β-spectrin or *mec-17*/tubulin acetyltransferase was mutated [21], [22], this suggests that microtubules confer resistance of touch neurites to deformation imposed by constant muscle activity. When this structural resilience is compromised, touch neurons are likely to degenerate presumably in a wear-and-tear fashion induced by the animal's constant movements. Supporting this notion, commissural axons in *unc-70* mutants also increasingly break as the animals grow and move, and paralysis of the animals prevents axon interruption [39]. Unlike the *mec-17* mutant, in which defective tubulin acetylation was associated with abnormal microtubule protofilament number, tubulin acetylation and microtubule protofilaments were unaffected in the *mec-12(gm379)* mutant. We hypothesize that the G416E substitution may alter the association of microtubule-binding proteins with microtubules, changing the stability or structure of microtubule lattice and rendering the neurites susceptible to mechanical strain. It would be interesting to test whether any of the tubulin mutations found in human diseases generates similar axon defects and could also be ameliorated by reduced activity of neighboring musculature. The molecular mechanism by which G416E mutant microtubules cause axon degeneration awaits future investigation.

## Materials and Methods

### *C. elegans* mutants, transgenes and RNAi

Strains were cultured as described [40]. The following alleles were used in this study: N2 (Bristol strain), CB4856, *LG I: dhc-1(or283ts), unc-54(e190); LG II: unc-104(rh43); LG III: mec-12(e1607)* (a gift from Martin Chalfie, Columbia University), *mec-12(tm5083), mec-12(gm379); LG V: sid-1(pk3321); him-5(e1490); LG X: mec-7(ok2152)*. Transgenes used in the current study are: *jsIs37(Pmec-7::SNB-1::GFP)/IV*, *jsIs219(Psng-1::SNG-1::GFP)/II, jsIs821(Pmec-7::GFP::RAB-3)/X*, *jsIs973(Pmec-7:mRFP)/III*, *jsIs1111(Pmec-4::UNC-104::GFP)*, *jsIs1238(Pmec-7::SYD-2::GFP)* (*jsIs821*, *jsIs973*, *jsIs1111*, and *jsIs1238* are gifts from Michael Nonet, Washington University), *juIs76(Punc-25::GFP)/II*, *Punc-17::RFP/V* (a gift from Joshua Kaplan, Massachusetts General Hospital), *orIs17[Pdhc-1::GFP::DHC-1, unc-119(+)]* (a gift from Bruce Bowerman, University of Oregon), *otIs118(Punc-33::GFP)/IV*, *uIs71(Pmec-18::SID-1, Pmyo-2::mCherry)*, *zdIs5[Pmec-4::GFP, lin-15(+)]/I*, *twnEx8(Pmec-7::TOMM20::mCherry, Pmyo-2::gfp)* (“*Pmec-7::mito::mCherry”*), *twnEx40(Pmec-7::GFP::EBP-2, Pdpy-30::dsRed)*, *twnEx42[Pmec-7::dhc-1(RNAi), Pmyo-2::GFP]*, *twnEx55[Pmec-7::MEC-12(G416E, E417G), Pdpy-30:: NLS::dsRed]*, *twnEx73[Pmec-7::MEC-12(G416E)* 5 ng/µl, *Pdpy-30::NLS::dsRed]*, *twnEx74[Pmec-7::MEC-12(G416D), Pdpy-30:: NLS::dsRed]*, *twnEx75[Pmec-7::MEC-12(G416Q), Pdpy-30:: NLS::dsRed]*, *twnEx76[Pmec-7::MEC-12(G416A), Pdpy-30:: NLS::dsRed]*, *twnEx77[Pmec-7::MEC-12(G416K), Pdpy-30:: NLS::dsRed]*, *twnEx78[Pmec-7::MEC-12(G416R), Pdpy-30:: NLS::dsRed]*, *twnEx79[Pmec-7::MEC-12(E414G, G416E), Pdpy-30:: NLS::dsRed]*, *twnEx80[Pmec-7::MEC-12(E415G, G416E), Pdpy-30:: NLS::dsRed]*, *twnEx88[Pmec-7::MEC-12(G416E)* 10 ng/µl, *Pdpy-30::NLS::dsRed]*, *twnEx89[Pmec-7::dhc-1(RNAi), Pdpy-30:: NLS::dsRed]*, *twnEx98[Punc-119::MEC-12, Punc-119::MEC-7, unc-119(+)]*, *twnEx99[Punc-119::MEC-12(G416E), Punc-119::MEC-7, unc-119(+)]*, *Ex(Punc-104::UNC-104::GFP)*, *Ex(Punc-104::UNC-104::mRFP)* (both from Oliver Wagner, National Tsing-Hua University, Taiwan). *mec-12(e1607)* is a G to A point mutation at nucleotide 430 of *mec-12* cDNA, resulting in glycine to serine mutation at amino acid 144. Germ line transformation was performed by microinjection of purified DNA of interest as described [41].

### EMS screen identification of *gm379*

Initially, an EMS mutagenesis screen was performed in the *zdIs5; cwn-1(ok546)* animals to identify mutations that cause synthetic polarity defects of the touch neurons [42]–[44]. While causing no defects in neuronal polarity, *gm379* was recovered due to its prominent axonal defects. The *cwn-1* mutation was then removed from the mutant. The SV transport defects, SV mistargeting and axonal swellings were all independent of the *cwn-1(ok546)* mutation in the background, and the *cwn-1* mutant displayed none of the *gm379* phenotypes.

### Single nucleotide polymorphism (SNP) mapping

Single nucleotide polymorphism mapping was performed as described [45]. *zdIs5* was included in this mapping to assist the identification of the homozygous *gm379* mutants. In brief, male animals of the Hawaiian strain CB4856 were crossed to *zdIs5; gm379*, and *gm379* homozygotes were later recovered from F2 progeny. F3 animals from individually cloned F2 animals were washed off plates and genomic DNA extracted by proteinase K treatment. To map *gm379*, we selected 48 SNPs from the 5 autosomes and the sex chromosome, and semi-quantitatively determined the ratio of N2/Hawaiian SNP for each locus using the restriction enzyme *Dra*I. Our SNP mapping located *gm379* to a region between -12 and +7 MU of Chromosome III, a region that contains the *mec-12* locus.

### RNAi experiments

Feeding RNAi was performed as described [46], with 1 mM IPTG pre-induction for 2 hours. For touch neuron-specific RNAi, we used *jsIs973(Pmec-7::mRFP) mec-12(gm379); sid-1(pk3321); jsIs821(Pmec-7::GFP::RAB-3); uIs71(Pmec-18::SID-1, Pmyo-2::mCherry)* animals [47]. The only RNAi-sensitive cells in the *sid-1; uIs71* background are the six touch receptor neurons. Five L4 animals were placed on the RNAi plates and cultured at 20°C. The F1 progeny of were then transferred to another freshly prepared RNAi plates at L4, and their progeny (F2) scored for axon and synaptic vesicle phenotypes. Each RNAi experiment was repeated three times to confirm the results. Efficiency of feeding RNAi against neuronal genes in this genotype was confirmed by *mec-12* RNAi, which resulted in 55% animals losing the PLM branch (n = 22), with control RNAi having no effects (0%, n = 27). This penetrance was similar to what was observed in the *mec-12(e1607)* null mutant (58%, n = 60). Additional RNAi control included *mec-7* and *rho-1* and all showed results comparable with mutant analysis.

### Molecular biology and plasmid construction

Cloning and construction of plasmids were performed with standard molecular biology techniques. All expression constructs in the *twnEx* series transgenes were in the pPD95.77 Fire vector backbone, which contains the *unc-54* 3′-UTR for optimized expression in *C. elegans*. Primer sequence information is available upon request.

### Scoring of ALM and PLM axon defects

Neurite swelling of ALM and PLM was scored in live animals with the integrated GFP reporter *zdIs5(Pmec-4::GFP)*, which is expressed in the six mechanosensory neurons: ALMs, PLMs, AVM and PVM. Beading is defined as oval or round swelling along primary axons. Neurite swelling is defined as triangular protrusion or looping of axonal membrane. Neurodegeneration is define as swelling and round-up of the neuronal soma with neurite interruption, thinning and large beading formation. To characterize the evolution of neurite defects in mutants, wild type and *gm379* animals were synchronized by hatching and arresting early L1 in M9 at 20°C. Animals were then allowed to feed on regular E. coli plates with axon morphology scored at different time points (6 hr, 12 hr, 24 hr, 36 hr, 48 hr, and 60 hr post hatching) that correspond to distinct larval and adult stages. Because *unc-54* animals are defective in egg laying and die from progeny hatched inside their bodies, 5-fluoro-2′-deoxyuridine (FUdR) was added to the plate at the final concentration of 50 µM to stop progeny production. FUdR was applied to the *mec-12(gm379)* mutant and the wild type in experiments where *unc-54* was also tested.

### Synaptic vesicle scoring

Synaptic vesicles in the touch neurons were visualized with the integrated GFP reporter *jsIs821(Pmec-7::GFP::RAB-3)* which labels synaptic vesicles in the six touch neurons. The authenticity of synaptic vesicle defects in the *gm379* mutant was confirmed with another GFP reporter, *jsIs37(Pmec-7::SNB-1::GFP)*. Touch neurons were simultaneously labeled by the RFP reporter *jsIs973(Pmec7::mRFP)*. Animals were synchronized and *jsIs821*-labeled synaptic vesicles quantified at distinct developmental stages. Images were acquired using the 63x Carl Zeiss Apochromat objective and the Zeiss AxioImager M2 imaging system. Because the posterior PLM process was very thin (less than 0.5 µm, see Figure S2B and S2C), we did not take confocal z-axis image stacks for pixel quantification. Pixel density was derived using ImageJ by quantifying total pixel number divided by the area marked by the neuronal marker *jsIs973*. We excluded the neuronal nucleus when quantifying pixel density of the soma. For Figure 5G, because the UNC-104-overexpression array carries mCherry fused to UNC-104, which may complicate determination of neurite area by the *jsIs973* marker, we decided to quantify total pixel number of fluorescence on the entire PLM posterior process. Mistargerted SVs often formed GFP::RAB-3 aggregates of variable size, and we therefore did not quantify GFP punctum number or individual punctual intensity. The length of the PLM posterior process was measured by the software Axio Vision Rel. 4.8. The distance of synaptic vesicle distribution was determined as the fraction of the PLM posterior process marked with synaptic vesicle GFP. All image quantification was done blind to avoid bias.

### Electron microscopy

Worms were high pressure frozen in either a Bal-Tec HPM 010 (Bal-Tec AG, Liechenstein) or Leica HMP 100 (Leica Microsystems, Vienna) high pressure freezer and freeze substituted in 1% osmium tetroxide and 0.1% uranyl acetate in acetone over a period of 2 hours by the SQFS method of McDonald and Webb [45]. Infiltration of Epon epoxy resin was carried out by 15 minute incubations in 25, 50, and 75% acetone-resin mixtures on a rocker, then three 15 minute incubations in pure resin. Polymerization of resin was for 2 hours in a 100°C oven. Sections of 70 nm thickness were post-stained with 2% uranyl acetate in 70% methanol for 4 minutes and lead citrate (Reynolds, 1963) for 2 minutes. Images were viewed on a Tecnai 12 (FEI Inc., Hillsboro, OR, USA) transmission electron microscope operating at 120 kV, and images recorded with a Gatan Ultrascan 1000 CCD camera (Gatan Inc., Pleasanton, CA, USA). Some high magnification views of microtubule were taken out of focus in order to highlight protofilament patterns [48], [49].

### Microtubule polarity by EBP-2::GFP dynamic imaging

EBP-2 comets were barely visible in wild type touch neurons, which could be attributed to the very stable microtubule structures in these cells. Therefore we devised an assay in which low-dose (0.125 mM) colchicine was applied to the worms to generate a moderate level of microtubule perturbation. L2 worms with *twnEx40(Pmec-7::EBP-2::GFP, Pmyo-2::GFP)* were grown on colchicine-containing NGM plates for 8 hours, picked off the plates and imaged one hour later. Under such treatment, a significant percentage of touch neurons displayed variable degree of microtubule growth with EBP-2 comets. Imaging acquisition was performed with the Zeiss AxioImager M2 imaging system.

### Immunostaining

Worm immunostaining was performed as described [50]. Briefly, mixed-stage animals were flash-frozen in liquid nitrogen and fixed in 2% paraformaldehyde on ice for at least 4 hours, permeabilized by Tris-Triton, and subjected to series of reduction and oxidation by sequential β-mercaptoethanol, dithiothreitol (DTT) and hydrogen peroxide treatment in 1% borate base buffer, and stained with primary antibodies in PBST-A. The following primary antibodies were used in this study: 6-11B-1 (mouse monoclonal anti-K40 acetylated α-tubulin, 1∶500, Santa Cruz Biotech), GT335 (mouse monoclonal anti-polyglutamylated tubulin, 1∶100, Enzo Life Sciences), rabbit polyclonal anti-detyrosinated tubulin (1∶200, Millipore), YL1/2 (rat monoclonal anti-tyrosinated tubulin, 1∶200, Santa Cruz Biotech), and rabbit polyclonal anti-GFP (1∶250, Santa Cruz Biotech). Secondary antibodies are goat anti-rabbit, goat anti-rat or goat anti-mouse IgG conjugated with Alexa488 or Alexa568 used at 1∶100 (Molecular Probes). Animals were counterstained with DAPI at 1∶1000 diluation in 2% n-propylgallate (NPG) and observed with the Zeiss AxioImager M2 imaging system. For fluorescence confocal microscopy, L4 to young adult hermaphrodite animals were anesthetized with 1% sodium azide, mounted on agar pad, and observed under Zeiss LSM700 confocal imaging system.

### Purification of DHC-1 MTBD

A fragment of the microtubule-binding domain (MTBD, amino acids 3207-3372) of *C. elegans* DHC-1 was cloned into the KpnI site of the pET30α vector with the primers: 5′ GGTACCCTCGCAGAGCAGCTGAAG 3′ (forward) and 5′ GGTACCTTATTCCTGGGTCTTCTTTGCAGC 3′ (reverse), and tagged at the N-terminus with 6xHis. Expression in *E. coli* was induced by 0.5 mM IPTG at 16°C for 4 hours, for avoiding protein aggregate formation in subsequent steps of purification. Bacterial pellet was collected by centrifugation and resuspended in the lysis buffer containing 50 mM NaH_2_PO_4_, 500 mM NaCl, 10 mM imidazole, 0.1% lysozyme, protease inhibitor cocktail and were homogenized by sonication. Cell extract was centrifuged at 15,000 g for 30 min at 4°C and MTBD was purified by passing the supernatant through HisPur™ Ni-NTA resin (Thermo Fisher Scientific, Walthem, USA). Purified MTBD was dialyzed in HEPES buffer (80 mM HEPES pH 7.0, 2 mM MgCl_2_, 0.5 EGTA) for microtubule binding protein spin-down assay.

### Microtubule sedimentation assay with worm protein extract

The microtubule sedimentation assay was performed as described with modifications [51]. In brief, *unc-119; twnEx98[Punc-119::MEC-12, Punc-119::MEC-7, unc-119(+)]* and *unc-119; twnEx99[Punc-119::MEC-12(G416E), Punc-119::MEC-7, unc-119(+)]* transgenic animals were grown to gravid adults on standard NGM plates. Animals were collected by washing and centrifugation in 0.1 M PIPES (pH 6.94), 4.0 mM MgCl_2_, 5 mM EGTA, 0.1 mM EDTA, 0.9 M glycerol, 1 mM PMSF, and 1 mM DTT (PMEG) at 4°C and resuspension in cold PMEG with protease inhibitor cocktail. Worms were then manually homogenized and centrifuged at 20,000xg for 45 min and the pellet discarded. The supernatant was centrifuged at 150,000xg for 60 min and the pellet discarded. The translucent supernatant was then supplemented with 2 mM GTP, 10 pM taxol, 1 U/ml hexokinase, 50 mM glucose, and 25–50 nm AMP-PNP and incubated on ice for 90 min for microtubule polymerization. Microtubule polymers were sedimented through 20% sucrose cushion made in PMEG with10 pM taxol by centrifugation at 20,000xg for 90 min. The pellet was resuspended in 1 ml PMEG containing 10 nM taxol and 50 mM NaCl. Microtubules and associated proteins were pelleted at 20,000xg for 40 min and dissolved in water for further analysis.

### In vitro microtubule binding protein spin-down assay

*In vitro* microtubule sedimentation assay was performed by using microtubule binding protein spin-down assay kit (Cytoskeleton, Denver, USA). In brief, microtubules were synthesized from purified bovine tubulins and incubated with purified MTBD at room temperature for 30 minutes. Microtubule-associated proteins were pelleted in sucrose cushion by centrifugation at 20000xg, resuspended and analyzed by SDS-PAGE. Coomassie blue-stained signals of tubulins and MTBD were quantified using ImageJ. The signal intensity of MTBD was normalized to respective tubulin signals.

### Western blot analysis

Protein lysate from microtubule sedimentation assay was boiled with SDS lysis buffer and separated by SDS-PAGE (6% acrylamide). Proteins were transferred to a nitrocellulose membrane and probed with mouse anti-UNC-104 monoclonal antibody (1∶60, a gift from Dr. S. Koushika) [52], rabbit anti-DHC-1 polyclonal antibody (1∶200, a gift from Dr. P. Gonczy) [30] or 6-11B-1 (1∶1000, Santa Cruz Biotech) for acetylated α-tubulin, followed by HRP-based chemiluminescence detection. Signal intensity was quantified using ImageJ. All experiments were repeated at least three times. To quantify the amount of DHC-1 or UNC-104 co-sedimented with microtubules, the pixel intensity of individual bands was first quantified using ImageJ. We first normalized the amount of bound DHC-1 or UNC-104 relative to sedimented microtubules in respective experiments. Next, we normalized the DHC-1/microtubule values to the averaged UNC-104/microtubule value, and data from five independent experiments were expressed as a fold change relative to the UNC-104/microtubule ratio.

## Supporting Information

Figure S1 Transport Defects of Active Zone Proteins in the *gm379* Mutant. (A) A schematic diagram of the *C. elegans* ALM and PLM neurons and their synapses. The “+” and “−” signs indicate the dominant microtubule orientation in the anterior ALM and PLM processes. (B-E, B′-E′) Vesicles containing the active zone protein SYD-2 were visualized and quantified in live animals with *jsIs1238(Pmec-7::SYD-2::GFP)* for the nerve ring synapses (B, B′), the ALM process (C, C′), the PLM synapses in the ventral nerve cord (D, D′) and the PLM process (E, E′). Arrows indicate SYD-2 puncta. (F) SYD-2 abundance measured by GFP quantification in respective locations of the touch neuron circuit. Scale bar  = 5 µm. **, *p*<0.005; ***, *p*<0.0001, Mann-Whitney U test.
(TIF)

Figure S2 Progressive Axonal Defects and Microtubule Ultrastructure of the Touch Neurons in the *gm379* Mutant. (A) Times indicate hours after synchronized, arrested early L1 larvae were placed on *E. coli* feeding plates. Beadings are focal enlargement of axons that appear rounded, whereas swellings are focal axonal lesions that expanded in diameter and often assumed a twisted triangular distortion in morphology. N>25 for animals scored at each time point. (B-D) Transmission electron micrographs showing microtubule organization of the PLM posterior process of the *gm379* mutant. (B) The characteristic 15-p giant microtubules were preserved in the PLM posterior process (small arrows) of the *gm379* mutant, although microtubule polymers were not as abundant as those in the ALM process. One microtubule polymer (big arrow) from a nearby unidentified cell, probably a neuron, could be seen to contain 11 protofilaments (magnified in the inset at upper left). The image was defocused on purpose to highlight the protofilament structure of the microtubule. Scale bar  = 50 nm or 10 nm (inset). (C-D) Images of the PLM posterior process from another *gm379* mutant animal. D is a magnified view of one of the microtubules in B. Scale bar  = 50 nm (C) or 10 nm (D).
(TIF)

Figure S3 SV Transport Defects in the *mec-12(e1607)* and the *mec-12(tm5083)* Null Mutants. (A) A schematic diagram of the touch neurons in *C. elegans*. (B-F, B′-F′, B″-F″) Epifluorescence images showing the distribution of SVs, represented by GFP::RAB-3 signal, in the wild type and the two *mec-12* null mutants. Both *mec-12(e1607)* and *mec-12(tm5083)* had severe defects in SV transport: GFP::RAB-3 signals were largely absent from the synapses in the nerve ring (B, B′, B″) and the ventral nerve cord (E, E′, E″), as well as the processes of the touch neurons (C, C′, C″), with SV accumulation in the neuronal soma (D, D′, D″ and F, F′, F″). However, SVs were not mistargeted to the PLM posterior process (F, F′, F″). Asterisks, ALM (D) or PLM (F) soma. Scale bar  = 5 µm.
(TIF)

Figure S4 Microtubule Post-Translational Modifications in Various Tubulin Mutants. (A-B) Mixed-stage animals were processed and stained with monoclonal antibodies for (A) acetylated microtubules (6-11B-1), tyrosinated tubulin (YL1/2), or (B) polyglutamylated microtubules (GT335). Only images of the adult animals were shown. Anterior is to the left. Immunoreactivity for acetylated microtubules was wild-type for *mec-12(gm379)*, mildly reduced for *mec-12(e1605)*, a partial loss-of-function allele, significantly reduced for *mec-7(ok2152)*, and absent for *mec-12(e1607)*. We did not detect de-tyrosinated or polyglutamylated microtubules in the touch neurons. GT335 stained sensory cilia (arrows) of amphid and phasmid neurons in the wild type and the *mec-12(gm379)* mutant. Asterisks, PLM cell bodies. Scale bar  = 5 µm. (C) Immunostaining of acetylated microtubules by 6-11B-1 monoclonal antibody in N2 (wild-type), *unc-119; twnEx98[Punc-119::MEC-12, Punc-119::MEC-7, unc-119(+)]*, and *unc-119; twnEx99[Punc-119::MEC-12(G416E), Punc-119::MEC-7, unc-119(+)]*. In N2, 6-11B-1 immunoreactivity was robust in the touch neurons, modest in the ventral nerve cord and the nerve ring, and undetectable in all other neurons or neurites. In animals with ectopic *mec-12* and *mec-7* expression, 6-11B-1 immunoreactivity could be detected in lateral nerves, commissures and lateral neurons, such as the SDQR. Cells normally with 6-11B-1 staining were labeled in white (the ALM and the AVM), and cells or neurites with ectopic 6-11B-1 immunoreactivity were marked in yellow.
(TIF)

Figure S5 *mec-7*/β-Tubulin Mutations Suppressed Axon Swelling and SV Mistargeting in the *mec-12(gm379)* Mutant. (A) Epifluorescence iamges showing axon swelling and SV mistargeting in the *mec-12(gm379)*, *mec-7(ok2152)*, and the *mec-12(gm379); mec-7* mutants. Anterior is to the left. Arrows indicate axon swellings (left panels, ALM) or SV mistargeting (right panels, PLM). Asterisks mark the ALM (left panels) and the PLM (right panels) cell bodies. (B) Quantification of axon defects or SV mistargeting in the mutants. Scale bar  = 5 µm. ***, *p*<0.0001, two proportion *z* test.
(TIF)

Figure S6 Microtubule Polarity of the PLM Processes. (A) Experimental procedures of EBP-2::GFP imaging with low-concentration colchicine (0.125 mM). Embryos of transgenic animals with *twnEx40(Pmec-7::EBP-2::GFP)* were allowed to hatch and grow on colchicine-containing NGM plates with food for 24 hours. These L2 larvae were then picked off the colchicine plates and placed on normal NGM plates with food for 2 to 4 hours to wash out colchicine and allow microtubules to re-polymerize. (B) Representative kymographs and quantification of EBP-2::GFP dynamic imaging in the PLM anterior and posterior processes of the wild type and the *mec-12(gm379)* mutant. Anterior is to the left. EBP-2 GFP comets that move distally away from the soma mark growing microtubules with their plus ends out. By contrast, EBP-2 GFP comets that move towards the soma label microtubules with their minus ends out.
(TIF)

Figure S7 Effects of UNC-104 Overexpression or *dhc-1* RNAi on SV Targeting in the *mec-12(gm379)* Mutant. (A) Epifluorescence images showing SV distribution in the ALM of the wild type and the mutants. SVs were visualized with *jsIs821(Pmec-7::GFP::RAB-3)*. Arrowheads, SVs. Asterisks, ALM soma. (B) SV quantification of the nerve ring synapse and the soma of the ALM neurons. Scale bar  = 5 µm. **, *p*<0.005; ***, *p*<0.0005, Mann-Whitney U test.
(TIF)

Figure S8 Effects of transgenic *dhc-1* RNAi on SVs in the PLM neuron. (A) Quantification of SVs mistargeted to the PLM posterior process in the *mec-12(gm379)* with or without *twnEx89[Pmec-7::dhc-1(RNAi)]* in the PLM, compared to non-transgenic animals. Statistical significance was assayed by Mann-Whitney U test. N.S., not significant. (B) Effects of two *dhc-1* RNAi arrays, *twnEx42* and *twnEx89*, on SVs in the PLM soma or at the PLM synapses. Reporters for SVs are *jsIs37(Pmec-7::SNB-1::GFP)* or *jsIs821(Pmec-7::GFP::RAB-3)*.
(TIF)

Figure S9 Original Coomassie blue-stained SDS-PAGE gel images of microtubule sedimentation with wild-type MTBD or MTBD(D3323K). All three independent experiments were shown. LC, loading control. Lanes from left to right represent serial 2-fold dilution of MTBD loading. Experiment 1 was shown in Figure 8A.
(TIF)

Video S1 A *mec-12(gm379)* adult hermaphrodite was partially immobilized with microbeads. The PLM neurite was imaged.
(AVI)
