# Supplementary figures and images for: Genetic Analysis of a Novel Tubulin Mutation That Redirects Synaptic Vesicle Targeting and Causes Neurite Degeneration in C. elegans
Source: PLoS Genet. 2014 Nov 13;10(11):e1004715. doi: 10.1371/journal.pgen.1004715 (PMC4230729; doi:10.1371/journal.pgen.1004715)

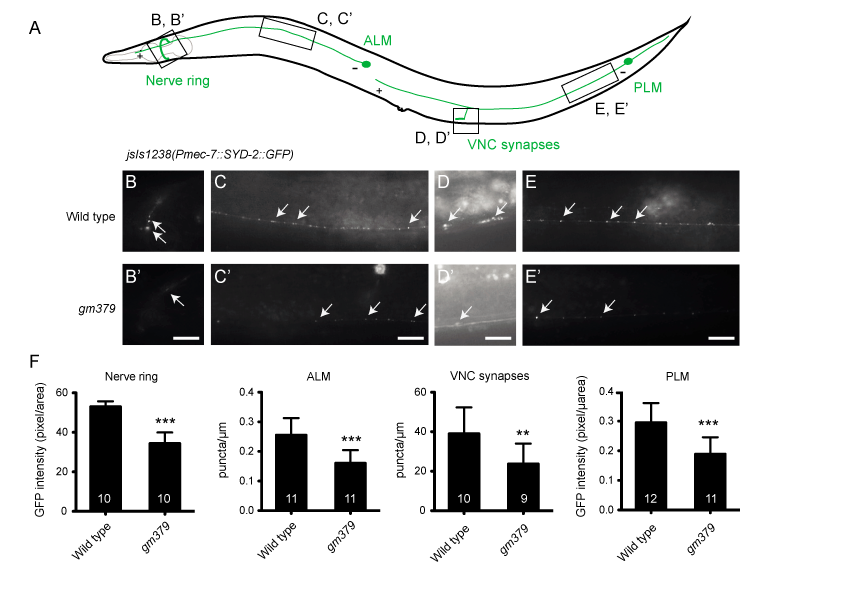

Supplement: Figure S1 — Transport Defects of Active Zone Proteins in the gm379 Mutant. (A) A schematic diagram of the C. elegans ALM and PLM neurons and their synapses. The “+” and “−” signs indicate the dominant microtubule orientation in the anterior ALM and PLM processes. (B-E, B′-E′) Vesicles containing the active zone protein SYD-2 were visualized and quantified in live animals with jsIs1238(Pmec-7::SYD-2::GFP) for the nerve ring synapses (B, B′), the ALM process (C, C′), the PLM synapses in the ventral nerve cord (D, D′) and the PLM process (E, E′). Arrows indicate SYD-2 puncta. (F) SYD-2 abundance measured by GFP quantification in respective locations of the touch neuron circuit. Scale bar = 5 µm. **, p<0.005; ***, p<0.0001, Mann-Whitney U test. (TIF) [file pgen.1004715.s001.tif]

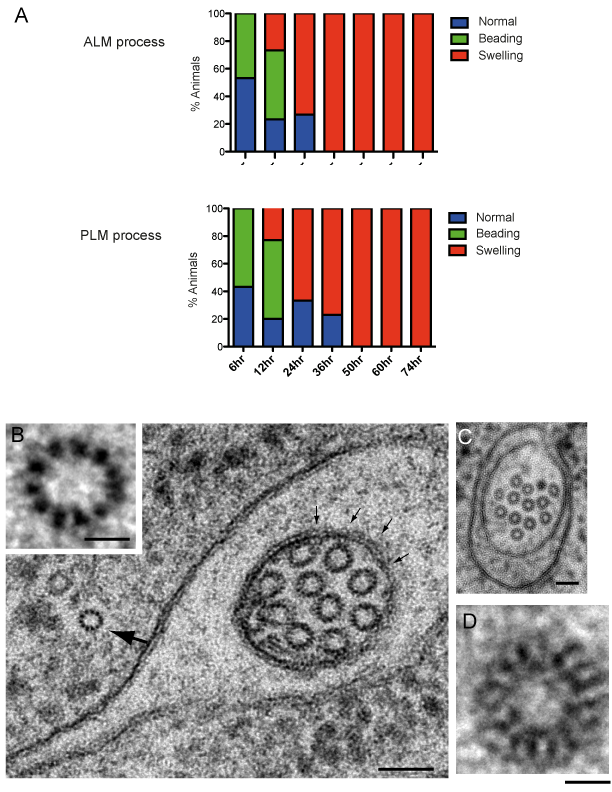

Supplement: Figure S2 — Progressive Axonal Defects and Microtubule Ultrastructure of the Touch Neurons in the gm379 Mutant. (A) Times indicate hours after synchronized, arrested early L1 larvae were placed on E. coli feeding plates. Beadings are focal enlargement of axons that appear rounded, whereas swellings are focal axonal lesions that expanded in diameter and often assumed a twisted triangular distortion in morphology. N>25 for animals scored at each time point. (B-D) Transmission electron micrographs showing microtubule organization of the PLM posterior process of the gm379 mutant. (B) The characteristic 15-p giant microtubules were preserved in the PLM posterior process (small arrows) of the gm379 mutant, although microtubule polymers were not as abundant as those in the ALM process. One microtubule polymer (big arrow) from a nearby unidentified cell, probably a neuron, could be seen to contain 11 protofilaments (magnified in the inset at upper left). The image was defocused on purpose to highlight the protofilament structure of the microtubule. Scale bar = 50 nm or 10 nm (inset). (C-D) Images of the PLM posterior process from another gm379 mutant animal. D is a magnified view of one of the microtubules in B. Scale bar = 50 nm (C) or 10 nm (D). (TIF) [file pgen.1004715.s002.tif]

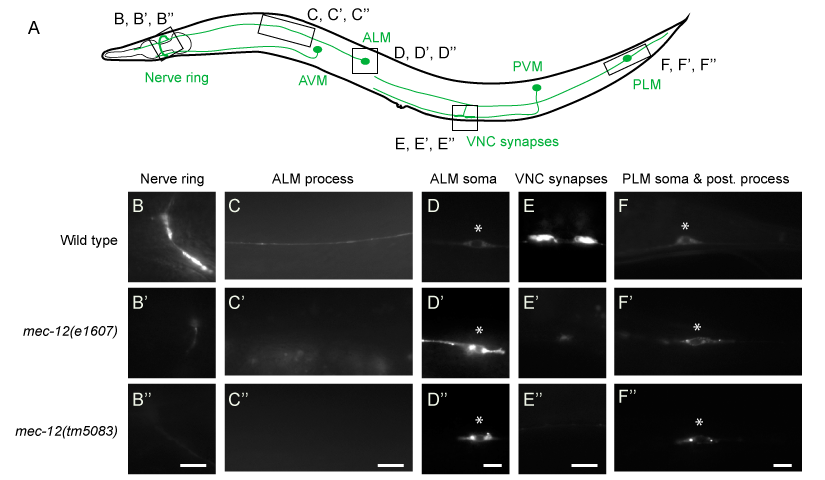

Supplement: Figure S3 — SV Transport Defects in the mec-12(e1607) and the mec-12(tm5083) Null Mutants. (A) A schematic diagram of the touch neurons in C. elegans. (B-F, B′-F′, B″-F″) Epifluorescence images showing the distribution of SVs, represented by GFP::RAB-3 signal, in the wild type and the two mec-12 null mutants. Both mec-12(e1607) and mec-12(tm5083) had severe defects in SV transport: GFP::RAB-3 signals were largely absent from the synapses in the nerve ring (B, B′, B″) and the ventral nerve cord (E, E′, E″), as well as the processes of the touch neurons (C, C′, C″), with SV accumulation in the neuronal soma (D, D′, D″ and F, F′, F″). However, SVs were not mistargeted to the PLM posterior process (F, F′, F″). Asterisks, ALM (D) or PLM (F) soma. Scale bar = 5 µm. (TIF) [file pgen.1004715.s003.tif]

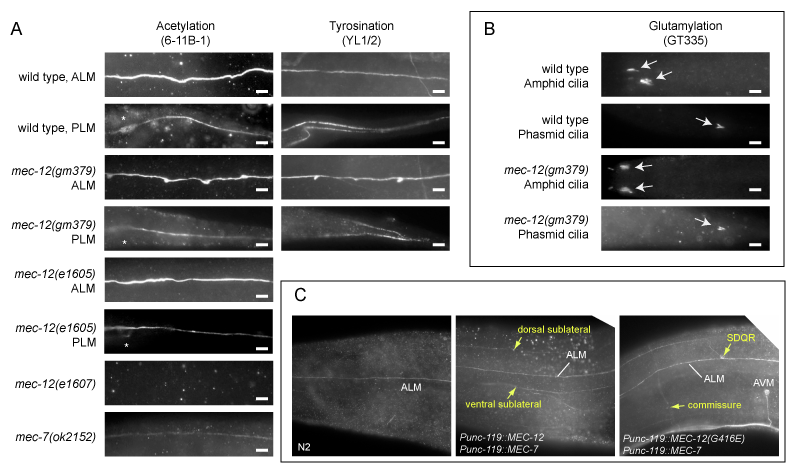

Supplement: Figure S4 — Microtubule Post-Translational Modifications in Various Tubulin Mutants. (A-B) Mixed-stage animals were processed and stained with monoclonal antibodies for (A) acetylated microtubules (6-11B-1), tyrosinated tubulin (YL1/2), or (B) polyglutamylated microtubules (GT335). Only images of the adult animals were shown. Anterior is to the left. Immunoreactivity for acetylated microtubules was wild-type for mec-12(gm379), mildly reduced for mec-12(e1605), a partial loss-of-function allele, significantly reduced for mec-7(ok2152), and absent for mec-12(e1607). We did not detect de-tyrosinated or polyglutamylated microtubules in the touch neurons. GT335 stained sensory cilia (arrows) of amphid and phasmid neurons in the wild type and the mec-12(gm379) mutant. Asterisks, PLM cell bodies. Scale bar = 5 µm. (C) Immunostaining of acetylated microtubules by 6-11B-1 monoclonal antibody in N2 (wild-type), unc-119; twnEx98[Punc-119::MEC-12, Punc-119::MEC-7, unc-119(+)], and unc-119; twnEx99[Punc-119::MEC-12(G416E), Punc-119::MEC-7, unc-119(+)]. In N2, 6-11B-1 immunoreactivity was robust in the touch neurons, modest in the ventral nerve cord and the nerve ring, and undetectable in all other neurons or neurites. In animals with ectopic mec-12 and mec-7 expression, 6-11B-1 immunoreactivity could be detected in lateral nerves, commissures and lateral neurons, such as the SDQR. Cells normally with 6-11B-1 staining were labeled in white (the ALM and the AVM), and cells or neurites with ectopic 6-11B-1 immunoreactivity were marked in yellow. (TIF) [file pgen.1004715.s004.tif]

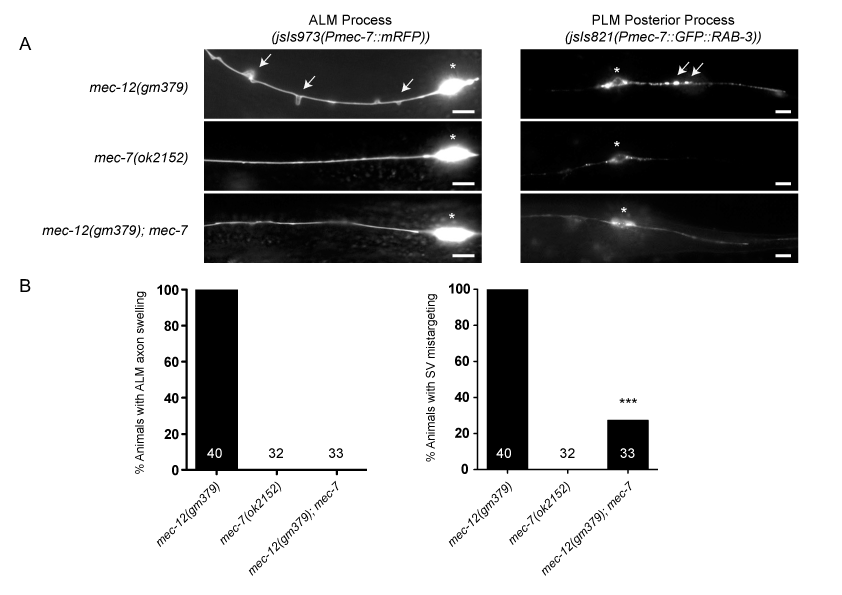

Supplement: Figure S5 — mec-7/β-Tubulin Mutations Suppressed Axon Swelling and SV Mistargeting in the mec-12(gm379) Mutant. (A) Epifluorescence iamges showing axon swelling and SV mistargeting in the mec-12(gm379), mec-7(ok2152), and the mec-12(gm379); mec-7 mutants. Anterior is to the left. Arrows indicate axon swellings (left panels, ALM) or SV mistargeting (right panels, PLM). Asterisks mark the ALM (left panels) and the PLM (right panels) cell bodies. (B) Quantification of axon defects or SV mistargeting in the mutants. Scale bar = 5 µm. ***, p<0.0001, two proportion z test. (TIF) [file pgen.1004715.s005.tif]

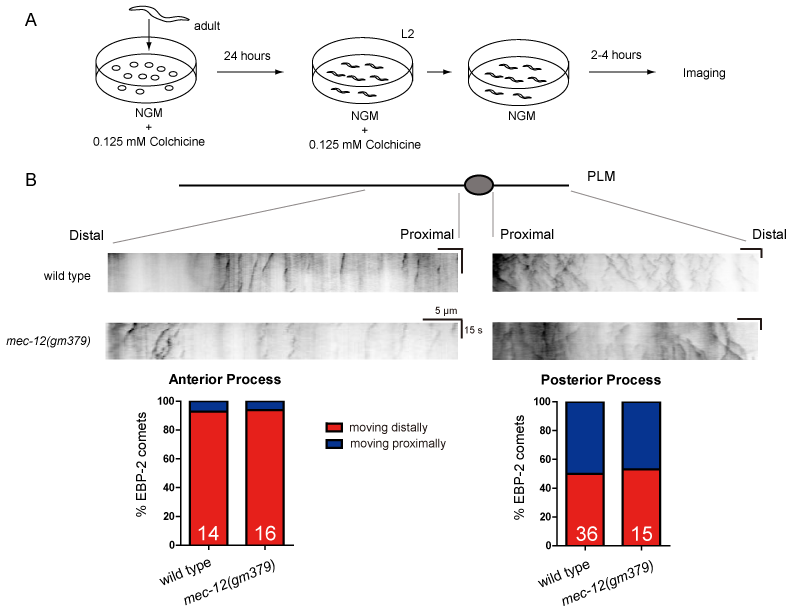

Supplement: Figure S6 — Microtubule Polarity of the PLM Processes. (A) Experimental procedures of EBP-2::GFP imaging with low-concentration colchicine (0.125 mM). Embryos of transgenic animals with twnEx40(Pmec-7::EBP-2::GFP) were allowed to hatch and grow on colchicine-containing NGM plates with food for 24 hours. These L2 larvae were then picked off the colchicine plates and placed on normal NGM plates with food for 2 to 4 hours to wash out colchicine and allow microtubules to re-polymerize. (B) Representative kymographs and quantification of EBP-2::GFP dynamic imaging in the PLM anterior and posterior processes of the wild type and the mec-12(gm379) mutant. Anterior is to the left. EBP-2 GFP comets that move distally away from the soma mark growing microtubules with their plus ends out. By contrast, EBP-2 GFP comets that move towards the soma label microtubules with their minus ends out. (TIF) [file pgen.1004715.s006.tif]

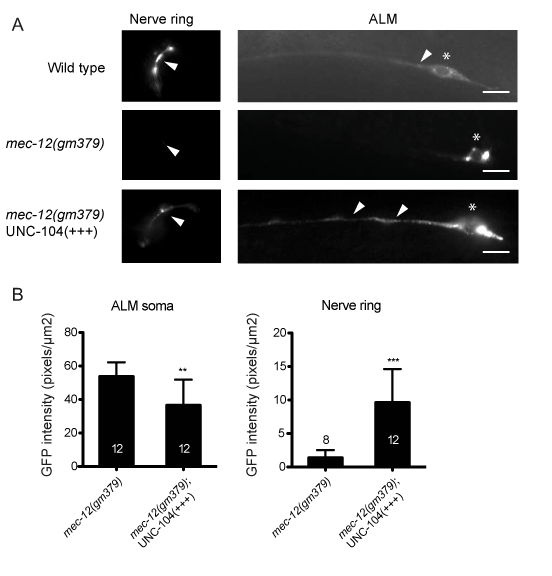

Supplement: Figure S7 — Effects of UNC-104 Overexpression or dhc-1 RNAi on SV Targeting in the mec-12(gm379) Mutant. (A) Epifluorescence images showing SV distribution in the ALM of the wild type and the mutants. SVs were visualized with jsIs821(Pmec-7::GFP::RAB-3). Arrowheads, SVs. Asterisks, ALM soma. (B) SV quantification of the nerve ring synapse and the soma of the ALM neurons. Scale bar = 5 µm. **, p<0.005; ***, p<0.0005, Mann-Whitney U test. (TIF) [file pgen.1004715.s007.tif]

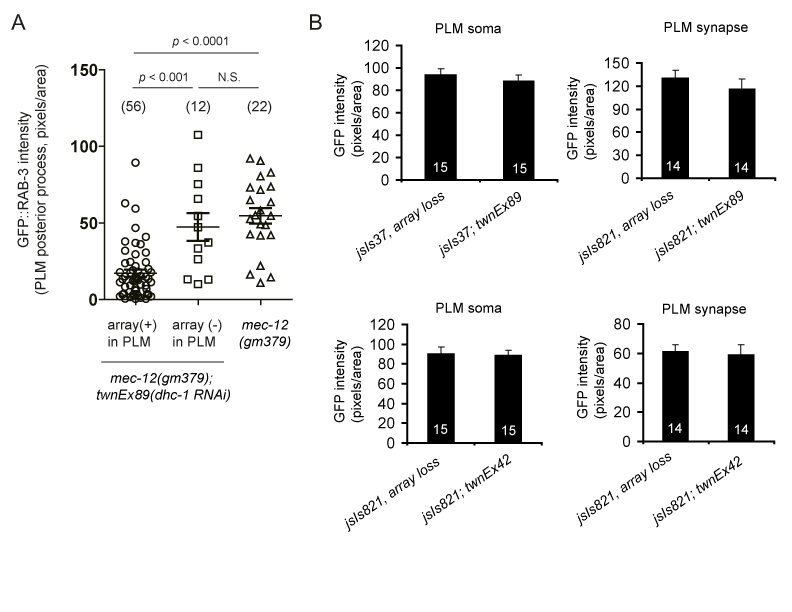

Supplement: Figure S8 — Effects of transgenic dhc-1 RNAi on SVs in the PLM neuron. (A) Quantification of SVs mistargeted to the PLM posterior process in the mec-12(gm379) with or without twnEx89[Pmec-7::dhc-1(RNAi)] in the PLM, compared to non-transgenic animals. Statistical significance was assayed by Mann-Whitney U test. N.S., not significant. (B) Effects of two dhc-1 RNAi arrays, twnEx42 and twnEx89, on SVs in the PLM soma or at the PLM synapses. Reporters for SVs are jsIs37(Pmec-7::SNB-1::GFP) or jsIs821(Pmec-7::GFP::RAB-3). (TIF) [file pgen.1004715.s008.tif]

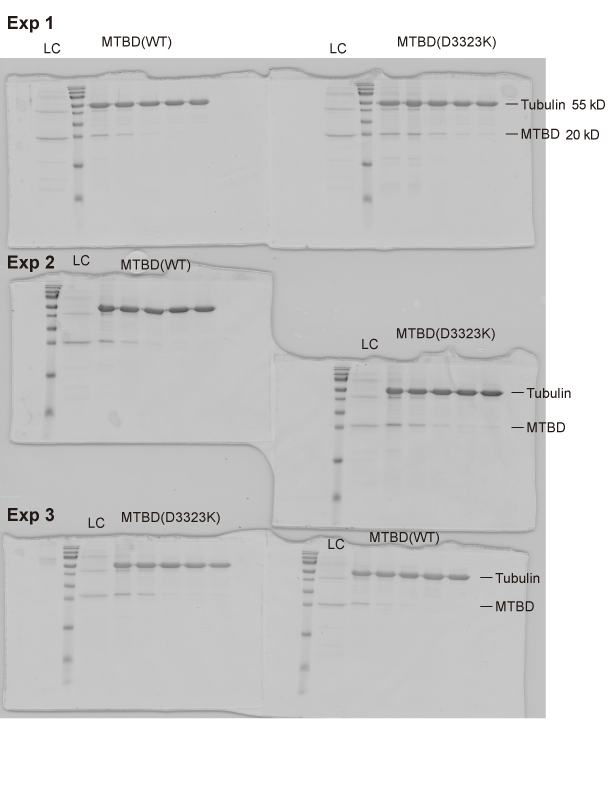

Supplement: Figure S9 — Original Coomassie blue-stained SDS-PAGE gel images of microtubule sedimentation with wild-type MTBD or MTBD(D3323K). All three independent experiments were shown. LC, loading control. Lanes from left to right represent serial 2-fold dilution of MTBD loading. Experiment 1 was shown in Figure 8A. (TIF) [file pgen.1004715.s009.tif]
